# Simultaneous quantification of proposed anti-malarial combination comprising of lumefantrine and CDRI 97–78 in rat plasma using the HPLC–ESI-MS/MS method: application to drug interaction study

**DOI:** 10.1186/s12936-015-0684-5

**Published:** 2015-04-22

**Authors:** Muhammad Wahajuddin, Sheelendra P Singh, Isha Taneja, Kanumuri SR Raju, Jiaur R Gayen, Hefazat H Siddiqui, Shio K Singh

**Affiliations:** Academy of Scientific and Industrial Research, New Delhi, India; Pharmacokinetics and Metabolism Division, CSIR- Central Drug Research Institute, Lucknow, 226031 Uttar Pradesh India; Analytical Chemistry Division, CSIR-Indian Institute of Toxicology Research, Lucknow, 226001 Uttar Pradesh India; Faculty of Pharmacy, Integral University, Lucknow, India

**Keywords:** Validation, Lumefantrine, Extraction, Malaria, Resistance, HPLC-ESI-MS-MS

## Abstract

**Background:**

Lumefantrine is the mainstay of anti-malarial combination therapy in most endemic countries presently. However, it cannot be used alone owing to its long onset time of action. CDRI 97–78 is a promising trioxane-derivative anti-malarial candidate that is currently being investigated as a substitute for artemisinin derivatives owing to their emerging resistance.

**Methods:**

In the present study, a sensitive, simple and rapid high-performance liquid chromatography coupled with positive ion electrospray ionization-tandem mass spectrometry (LC-ESI-MS/MS) method was developed for the simultaneous determination of lumefantrine and CDRI 97-78’s metabolite, 97–63, in rat plasma using halofantrine as an internal standard. Lumefantrine and 97–63 were separated on a Waters Atlantis C18 (4.6 × 50 mm, 5.0 μm) column under isocratic condition with mobile phase consisting of acetonitrile: methanol (50:50, v/v) and ammonium formate buffer (10 mM, pH 4.5) in the ratio of 95:5 (v/v) at a flow rate of 0.65 mL/min.

**Results:**

The method was accurate and precise within the linearity range 3.9-500 ng/mL for both lumefantrine and 97–63 with a correlation coefficient (r^2^) of ≥0.998. The intra- and inter-day assay precision ranged from 2.24 to 7.14% and 3.97 to 5.90%, and intra- and inter-day assay accuracy was between 94.93 and 109.51% and 96.87 and 108.38%, respectively, for both the analytes. Upon coadministration of 97–78, the relative bioavailability of lumefantrine significantly decreased to 64.41%.

**Conclusions:**

A highly sensitive, specific and reproducible high-throughput LC-ESI-MS/MS assay was developed and validated to quantify lumefantrine and CDRI 97–78. The method was successfully applied to study the effect of oral co-administration of lumefantrine on the pharmacokinetics of 97–78 in male *Sprague–Dawley* rats and *vice versa*. Co-administration of 97–78 significantly decreased the systemic exposure of lumefantrine.

**Electronic supplementary material:**

The online version of this article (doi:10.1186/s12936-015-0684-5) contains supplementary material, which is available to authorized users.

## Background

Artemether-lumefantrine (AL) is a first-line artemisinin combination therapy (ACT) recommended by WHO for the treatment of uncomplicated *Plasmodium falciparum* malaria [1]. The drugs are commercially available as a fixed dose combination in form of tablets, Coartem^®^ and Riamet^®^. The combination of a short-acting and a long-acting drug is apt for treatment of malaria cases since the short-acting partner kills most of the circulating parasites while the long-acting drug clears the remaining more slowly, thus preventing recrudescence. Lumefantrine (LUME) (previously known as benflumetol) was synthesized in the 1970s by the Academy of Military Medical Sciences in Beijing, China. It is a racemic fluorene derivative, named 2-dibutylamino-1-[2,7-dichloro-9-(4-chlorobenzylidene)- 9H-fluoren-4-yl]-ethanol [2,3]. It is the long-acting partner drug of the artemisinin derivative artemether, in this ACT. However, recently reports of artemisinin resistance have emerged from the Greater Mekong Subregion of Myanmar, Cambodia, Thailand, and Vietnam with increased parasite clearance times [1,4].

**Figure 1:**
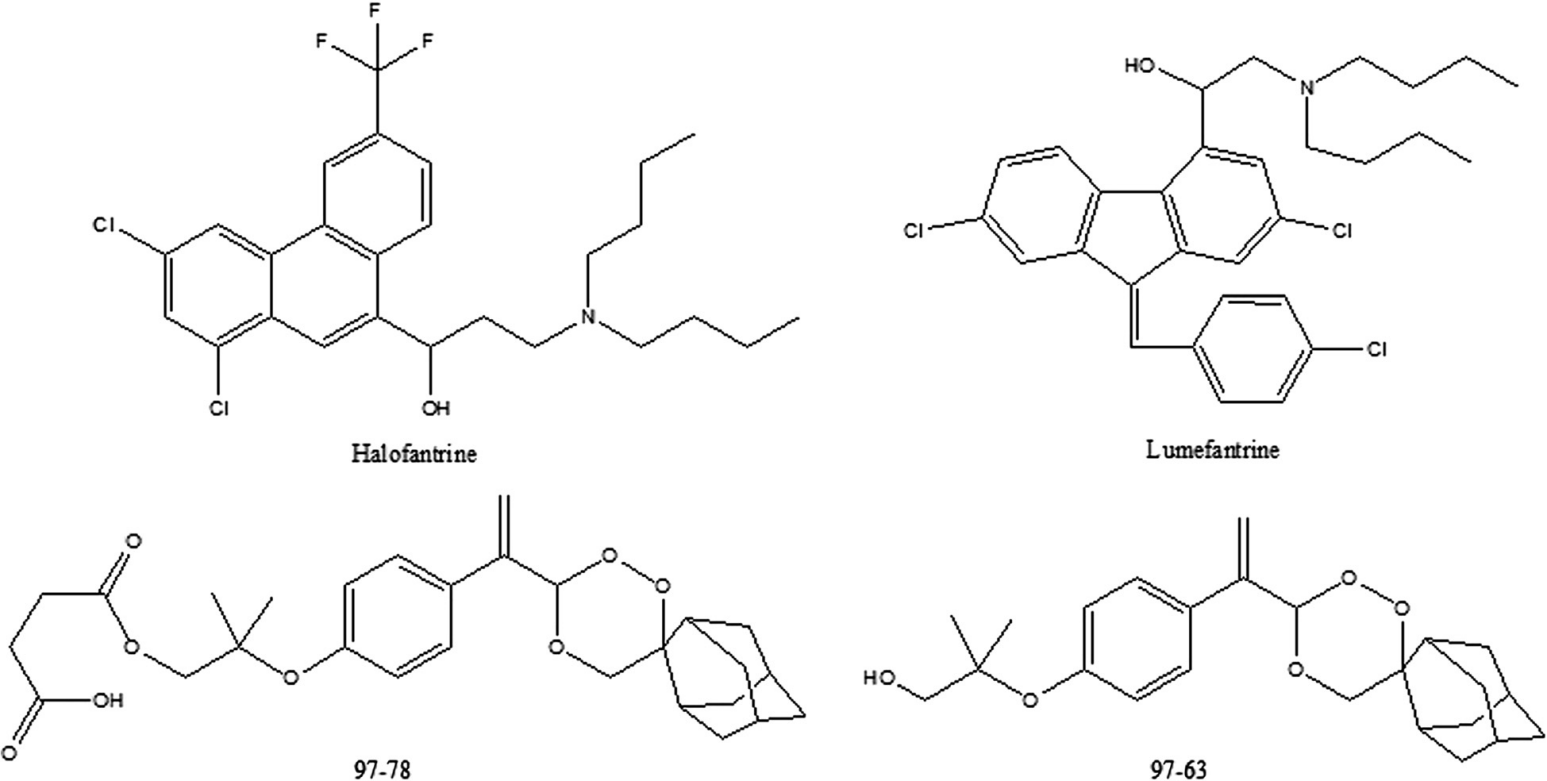
Structural representation of LUME, halofantrine, CDRI 97–78, and its metabolite 97–63.

Owing to the emerging resistance to artemisinin derivatives as well as their low oral bioavailability, there is the need to find novel anti-malarial agents that are devoid of such drawbacks and are more affordable to serve as an artemisinin alternative. To find a suitable fast-acting partner drug for LUME, CSIR-Central Drug Research Institute (India) (CDRI) has developed a series of trioxane anti-malarial compounds in its drug discovery programme [5,6]. One of the most promising compounds among these is CDRI candidate drug molecule 97–78 (Figure 1), which has been identified for development as a suitable alternative for artemisinin derivatives for use against drug-resistant *P. falciparum* and cerebral malaria cases. It is currently under Phase 1 clinical trials in licensing agreement with IPCA Pharmaceuticals Ltd, Mumbai, India. It is a water-soluble synthetic 1,2,4-trioxane derivative active in both rodent and monkey malaria models. This CDRI drug candidate has undergone preclinical efficacy, regulatory toxicity and pharmacological studies and has been found safe. Its Phase 1 safety trials have been completed in 50 volunteers and Phase 1 single dose pharmacokinetics in 16 volunteers [7]. *In vivo*, 97–78 gets rapidly and completely converted into its active metabolite 97–63 (Figure 1), which is quantified in the biological system [8].

The preclinical pharmacokinetics of LUME (Figure 1) has been established previously [9-11]. In the present report, a simple, sensitive and specific liquid chromatography is described coupled with positive ion electrospray ionization-tandem mass spectrometry (LC-ESI-MS/MS) method developed and validated for the simultaneous quantification of LUME and 97–63 in 100 μL rat plasma using halofantrine (Figure 1) as an internal standard (IS). The validated method was then applied to study the preclinical pharmacokinetic interaction of LUME and CDRI 97–78 combination to evaluate its prospects as a potential anti-malarial combination.

## Methods

### Chemicals and reagents

LUME and halofantrine (IS) were a generous gift from Ipca Laboratories Ltd, Mumbai, India. 97–78 and 97–63 were synthesized at the Medicinal Chemistry Division of Central Drug Research Institute, Lucknow, India. High performance liquid chromatography (HPLC) grade acetonitrile was purchased from Sisco Research Laboratories (SRL) Pvt Ltd, Mumbai, India. HPLC grade methanol was purchased from Thomas Baker Pvt Ltd, Mumbai, India. Ammonium formate and glacial acetic acid AR were purchased from E Merck Ltd, Mumbai, India. Sodium carboxy methyl cellulose (CMC) was purchased from Sigma Aldrich Ltd, St Louis, USA. Ultra-pure water was obtained from a Sartorius Arium 611 system. Heparin sodium injection IP (1,000 IU/mL, Biologicals E Ltd, Hyderabad, India) was purchased from local pharmacy.

### Animals and prerequisites

Blank, drug-free, plasma samples were collected from adult, healthy male *Sprague–Dawley* (SD) rats at the Division of Laboratory Animals (DOLA) of Central Drug Research Institute, Lucknow, India. Plasma was obtained by centrifuging the heparinized blood (25 IU/mL) at 2,000 × g for 10 min at 20°C. Prior approval from the Institutional Animal Ethics Committee (IAEC) was sought for maintenance, experimental studies, euthanasia, and disposal of carcass of animals.

### Instrumentation and chromatographic conditions

HPLC system consisting of Series 200 pumps and auto-sampler with temperature-controlled Peltier-tray (Perkin- Elmer Corp, Norwalk, CT, USA) was used to inject 10-μL aliquots of the processed samples on a Waters Atlantis C18 column (4.6 × 50 mm, 5.0 μm). The system was run in isocratic mode with mobile phase consisting of acetonitrile: methanol (50:50, v/v) and 10 mM ammonium formate buffer (pH = 4.5) in the ratio of 95:5 (v/v) at a flow rate of 0.65 mL/min. Mobile phase was duly filtered through 0.22 μm Millipore filter (Billerica, MA, USA) and degassed ultrasonically for 15 min prior to use. Separations were performed at room temperature. Auto-sampler carry-over was determined by injecting the highest calibration standard then a blank sample. 97–78 rapidly converted to 97–63 in plasma [8], hence, 97–63 was quantified instead of 97–78.

Mass spectrometric detection was performed on an API 4000 mass spectrometer (Applied Biosystems/MDS Sciex, Toronto, Canada) equipped with an API electrospray ionization (ESI) source. The ion spray voltage was set at 5,500 V. The instrument parameters: nebulizer gas, curtain gas, auxillary gas, and collision gas, were set at 40, 13, 50, and 10, respectively. Compounds parameters: declustering potential (DP), collision energy (CE), entrance potential (EP), and collision exit potential (CXP) were 80, 33, 10, 10 V, 50, 30, 4, 10 V, and 90, 33, 6, 8 V for LUME, 97–63 and IS, respectively. Zero air was used as source gas while nitrogen was used as both curtain and collision gas. The mass spectrometer was operated at ESI positive ion mode and detection of the ions was performed in the multiple reaction monitoring (MRM) mode, monitoring the transition of m/z 529 precursor ion [M + H]^+^ to the m/z 511.3 product ion for LUME, m/z 418.2 precursor ion [M + H]^+^ to the m/z 119.1 product ion for 97–63 and m/z 502 precursor ion [M + H]^+^ to the m/z 142.2 product ion for IS. Quadrupole 1 and quadrupole 3 were maintained at unit resolution and dwell time was set at 200 ms. Data acquisition and quantitation were performed using analyst software version 1.4.1 (Applied Biosystems/MDS Sciex Toronto, Canada).

### Preparation of standard and quality control samples

Primary stock solutions of LUME, 97–63 and IS were prepared by dissolving the compounds in acidified methanol (1% glacial acetic acid) to achieve desired concentration of 1 mg/mL. Working standard solutions of LUME and 97–63 were prepared by combining the aliquots of each primary stock solution, and diluting with methanol. A working stock solution of IS (50 ng/mL) was prepared by diluting an aliquot of primary stock solution with acetonitrile. All the stock solutions were stored at 4°C until analysis and were found to be stable up to six months. Calibration standards of LUME and 97–63 (3.9, 7.8, 15.6, 31.25, 62.5, 125, 250, and 500 ng/mL) were prepared by spiking 90 μL of pooled, drug-free, rat plasma with the appropriate working standard solution of the analytes (10 μL). Quality control (QC) samples were prepared by individually spiking control rat plasma at four concentration levels (3.9 ng/mL (lower limit of quantitation, LLOQ), 10 ng/mL (QC low), 100 ng/mL (QC medium) and 400 ng/mL (QC high)) and stored at −70 ± 10°C until analysis.

### Recovery

The extraction recovery of analytes through protein precipitation extraction procedure was determined by comparing the peak areas of pre-spiked extracted plasma standard QC samples (n = 6) to those of the post-spiked standards at equivalent concentrations. Recoveries of LUME and 97–63 were determined at three concentration levels, LLOQ, QC low and QC high concentrations: 3.9, 10 and400 ng/mL, whereas the recovery of the IS was determined at a single concentration of 50 ng/mL.

$$ Recovery\ \left(\%\right)=\frac{Concentration\  of\ pre- spiked\  sample}{Concentration\  of\  post- spiked\  sample}\times 100 $$

### Sample preparation

All frozen study samples and QC samples were thawed and allowed to equilibrate at room temperature prior to analysis. A simple protein precipitation method was followed for extraction of analytes from rat plasma. To 100 μL of plasma in a tube, 200 μL of IS solution (50 ng/mL in acetonitrile), was added and vortexed for 10 min followed by centrifugation for 10 min at 15,000 × g. The supernatant (200 μL) was separated and was injected onto analytical column.

### Validation procedures

#### Specificity and selectivity

The specificity and selectivity has been studied by using independent plasma samples from six different rats to investigate the potential interferences at the chromatographic peak region for analyte and IS, using the proposed extraction procedure and chromatographic-MS conditions.

#### Matrix effect

Pooled rat plasma from six different rats was used. The effect of rat plasma constituents over the ionization of LUME, 97–63 and IS was determined by comparing the responses of the post-extracted plasma standard QC samples (low and high) (n = 6) with the response of analytes from neat standard samples. The neat samples of equivalent concentrations were prepared by spiking equal volume of the working stock solutions in mobile phase. The matrix effect was calculated as:

$$ \% ME = \frac{Response\  of\  the\  post\hbox{-} extracted\  spiked\  sample}{Response\  of\  the\  equivalent\  neat\  standard\  sample}\times 100 $$

#### Calibration curve

The plasma calibration curve was constructed using eight calibration standards (3.9-500 ng/mL for LUME and 97–63) prepared by spiking 90 μL of pooled, drug-free rat plasma with the appropriate working standard solution of the analytes (10 μL).

### Precision and accuracy

The intra-day assay precision and accuracy were estimated by analysing six replicates at four different QC levels, i.e., 3.9, 10, 100, and 400 ng/mL, for LUME and 97–63. The inter-day assay precision was determined by analysing the four levels QC samples on three different runs. The criteria for acceptability of the data included accuracy within ±15% deviation from the nominal values and a precision of within ±15% relative standard deviation (RSD), except for LLOQ, where it should not exceed ±20% for accuracy as well as precision.

### Stability experiments

All stability studies were conducted at two concentration levels, i.e., QC low and QC high, using six replicates at each concentration levels. Replicate injections of processed samples were analysed up to 18 hrs to establish auto-sampler stability (AS) of analytes and IS at 4°C. The peak areas of analyte and IS obtained at initial cycle were used as the reference to determine the stability at subsequent points. The stability of LUME and 97–63 in the biomatrix during 6 hrs of exposure at room temperature in rat plasma (bench top (BT)) was determined at ambient temperature (25 ± 2°C). Freeze/thaw (FT) stability was evaluated up to three cycles. In each cycle, samples were frozen for at least 12 hrs at −70 ± 10°C. Freezer stability of both analytes in rat plasma was assessed by analysing the QC samples stored at −70 ± 10°C for at least 15 days. Samples were considered to be stable if assay values were within the acceptable limits of accuracy (i.e., ±15% deviation) and precision (i.e., ±15% RSD).

### Dilution integrity

The dilution integrity experiment was performed with an aim to validate the dilution test to be carried out on higher analyte concentrations (above the upper limit of quantification), which may be encountered during real subject samples analysis. Dilution integrity experiments were carried out by 20 times dilution of plasma samples containing 8,000 ng/mL of LUME and 97–63 with blank plasma to obtain samples containing 400 ng/mL (QC high)of LUME and 97–63.

### Application to interaction study

Study was performed in male Sprague–Dawley rats (n = 5, weight range 200–220 g). The rats were fasted overnight (14–16 hrs) prior to the experiment but given free access to water. Rats were divided into three groups (n = 5 each): two control groups (LUME, 10 mg/kg, oral, suspension in 0.25% sodium CMC and 97–78, 70 mg/kg, oral, suspension in 0.25% sodium CMC), and one co-administration group (70 mg/kg of oral 97–78 and 10 mg/kg of oral LUME). Blood samples (approximately 0.25 mL) were collected from the retro-orbital plexus into heparinized microfuge tubes at 0.25, 0.50, 1, 3, 6, 9, 11, 13, 15, 24, 48, 72, and 120 hrs post-dosing and plasma was harvested by centrifuging the blood at 15,000xg for 10 min and stored frozen at −70 ± 10°C until bio-analysis.

### Pharmacokinetic and statistical analysis

Plasma data were subjected to non-compartmental pharmacokinetics analysis using WinNonlin (version 5.1, Pharsight Corp, Mountain View, CA, USA). The observed maximum plasma concentration (C_max_) and the time to reach the maximum plasma concentration (T_max_) were obtained by visual inspection of the experimental data. Area under the plasma concentration-time curve from time zero to the last quantifiable concentration (AUC_0-t_) was calculated using linear trapezoidal rule. The total area under the plasma concentration–time curve from time zero to time infinity (AUC_0-∞_) was calculated as the sum of AUC_0-t_ and C_last_/k_el_, where, C_last_ represents the last quantifiable concentration and K_el_ represents the terminal phase rate constant. The apparent elimination half-life (t_1/2_) was calculated as 0.693/k_el_ and the k_el_ was estimated by linear regression of the plasma concentrations in the log-linear terminal phase. The clearance (Cl/F); where F represents the oral bioavailability, was calculated as dose/AUC, and the volume of distribution (Vd/F) was calculated as (Cl/F)/kel. The data is presented as a mean ± SD. The pharmacokinetic parameters were compared using Student’s *t* test. A P-value of <0.05 was considered significant. The relative bioavailability (RB %) was calculated as follows:

$$ Relative\  bioavailability\ \left(RB\%\right)=\frac{AUC\  coadimin}{AUC\  control}\times 100 $$

## Results

### LC-MS/MS optimization

It was important to optimize extraction technique, chromatographic conditions and mass spectrometry parameters to develop and validate a selective and rapid assay method for simultaneously quantitation of LUME, 97–63 and IS in rat plasma. Protein precipitation was chosen as the sample preparation method. Several organic solvents, including acetic acid, trichloroacetic acid, acetonitrile and methanol, were investigated as the precipitation extraction solvents. Acetonitrile was chosen because of higher extraction efficiency for LUME, 97–63 and IS, and much cleaner samples than other solvents. Several column types and chromatographic conditions were tested in order to develop a short, robust and sensitive analytical method. A short (4.6 × 50 mm, 5.0 μm) Waters Atlantis C18 column with mobile phase consisting of acetonitrile: methanol (50:50, v/v) and 10 mM ammonium formate buffer (pH 4.5) in the ratio of 95:5 (v/v) at a flow rate of 0.65 mL/min provided the best compromise between selectivity and speed of analysis. The overall analysis time was 6.0 min. No carry-over was observed, as indicated by the lack of LUME and 97–63 and halofantrine (IS) peaks in the blank sample.

Mass parameters were optimized by infusing standard analyte solution (100 ng/mL) into the mass spectrometer. In order to optimize ESI conditions for LUME, 97–63 and IS, quadrupole full scans were carried out in positive ion detection mode. During a direct infusion experiment, the mass spectra for LUME, 97–63 and IS revealed peaks at m/z 529, 418.2 and 502, respectively, as protonated molecular ions [M + H]^+^. Following detailed optimization of mass spectrometry conditions (provided in instrumentation and chromatographic conditions section) m/z 529 precursor ion [M + H]^+^ to the m/z 511.3 product ion for LUME, m/z 418.2 precursor ion [M + H]^+^ to the m/z 119.1 product ion for 97–63 and m/z 502 precursor ion [M + H]^+^ to the m/z 142.2 product ion for IS was used for the quantitation purpose.

### Recovery

The extraction recovery of the LUME and 97–63 ranged from 94.63 to 109.56%, and the extraction recovery of the internal standard was 93.26% (See Additional file 1).

### Validation procedures

The procedures were followed as per US FDA guidelines [12].

### Specificity and matrix effect

**Figure 2:**
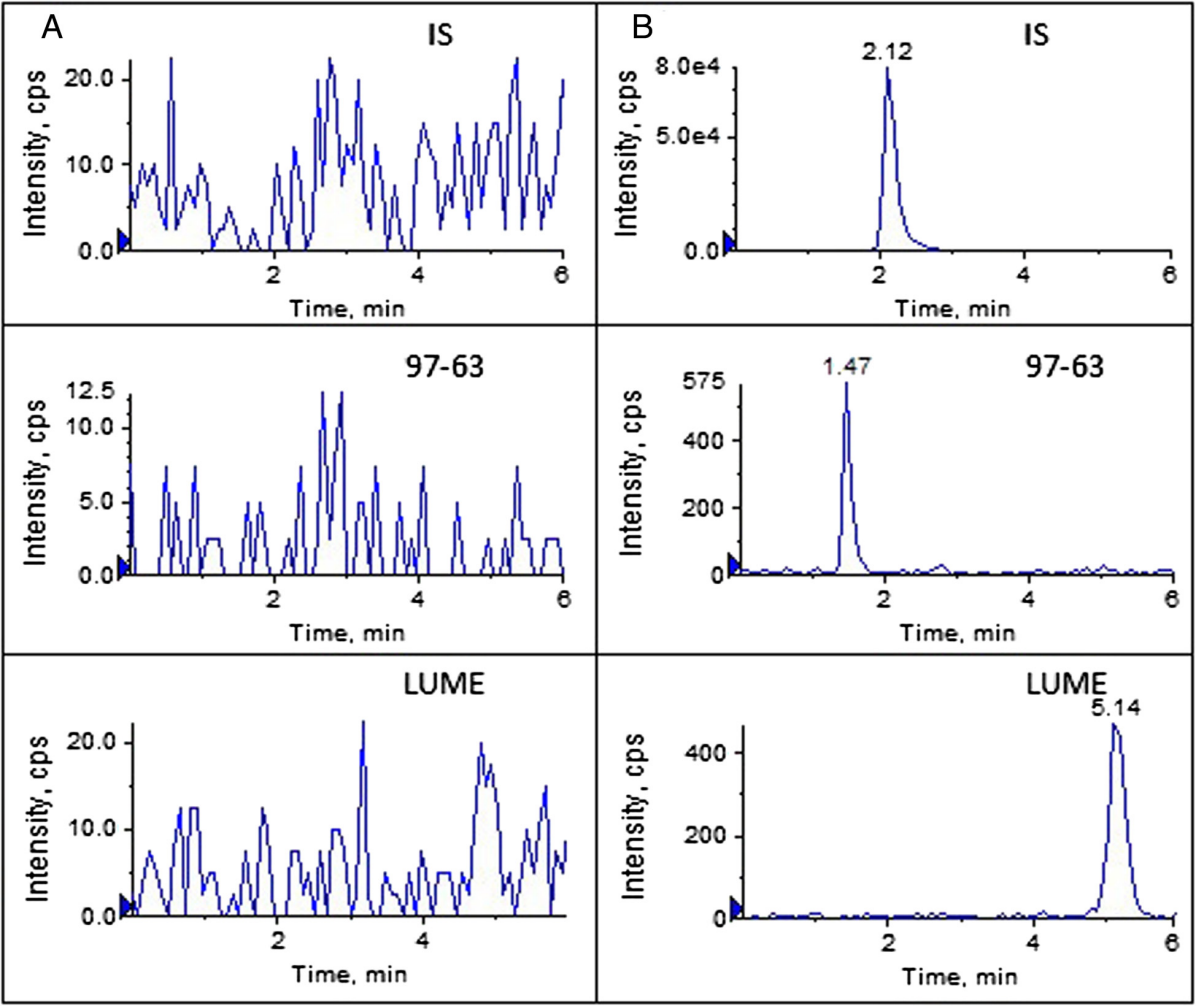
Typical MRM chromatograms of LUME and 97–63 in rat plasma. **(A)** a drug-free blank plasma, **(B)** drug-free plasma spiked with LUME and 97–63 at LLOQ (3.9 ng/mL) and IS.

In the present study, the specificity and selectivity has been studied by using independent plasma samples from six different rats. Figure 2 shows a typical chromatogram for the drug-free plasma (Figure 2A) and drug-free plasma spiked with LUME and 97–63 at LLOQ and IS (Figure 2B). As shown in Figure 2A, there is no significant interference from plasma found at retention time of either the analyte or the IS. The ion suppression or enhancement by plasma was less than 13.25% for the analytes and 18.5% for IS which demonstrated that the matrix effects do not cause quantitation bias. Therefore, matrix effect could be negligible under the experimental conditions (See Additional file 1).

### Calibration curve

The plasma calibration curve was constructed using eight calibration standards (3.9-500 ng/mL). The average regression (n = 3) was found to be ≥0.998. The percentage accuracy observed for the mean of back-calculated concentrations for three calibration curves was within 93.89 to 106.53, while the percentage precision values ranged from 1.39 to 9.00 for both the analytes.

### Accuracy and precision

Accuracy and precision data for intra- and inter-day plasma samples are presented in Tables 1 and 2. The assay values on both the occasions (intra- and inter-day) were found to be within the accepted variable limits.

**Table 1:** **Intra-day assay precision and accuracy for LUME and 97–63 in rat plasma (n = 6)**

	**LUME (ng/mL)**				**97-63 (ng/mL)**			
	**3.9**	**10**	**100**	**400**	**3.9**	**10**	**100**	**400**
**Day 1**								
Mean	3.9	9.6	97.4	387.3	4.1	10.2	104.0	405.2
SD	0.23	0.22	6.16	16.61	0.28	0.46	4.75	14.16
Precision (%)^a^	5.78	2.24	6.33	4.29	6.76	4.54	4.56	3.50
Accuracy (%)^b^	100.17	96.20	97.35	96.83	104.32	109.12	104.04	101.29
**Day 2**								
Mean	3.7	9.9	97.9	397.5	3.8	9.9	102.8	415.3
SD	0.27	0.55	6.93	11.74	0.23	0.53	3.99	9.35
Precision (%)^a^	7.14	5.51	7.07	2.95	6.01	5.27	3.88	2.25
Accuracy (%)^b^	95.90	99.47	97.92	99.38	99.40	106.52	102.78	103.83
**Day 3**								
Mean	3.8	9.5	103.0	415.0	3.8	10.3	104.3	386.5
SD	0.18	0.48	4.00	18.32	0.18	0.49	4.18	10.89
Precision (%)^a^	4.81	5.06	3.88	4.41	4.81	4.81	4.01	2.82
Accuracy (%)^b^	98.25	94.93	103.00	103.75	103.93	109.51	104.33	96.63

^a^ Expressed as % RSD = (SD/mean) × 100.

^b^ Calculated as (mean determined concentration/nominal concentration) × 100.

**Table 2:** **Inter-day assay precision and accuracy for LUME and 97–63 in rat plasma**

	**LUME (ng/mL)**				**97-63 (ng/mL)**			
	**3.9**	**10**	**100**	**400**	**3.9**	**10**	**100**	**400**
Mean^a^	3.8	9.7	98.1	399.9	4.0	10.2	103.7	402.3
SD	0.23	0.46	5.37	18.94	0.23	0.48	4.11	16.45
Precision (%)^b^	5.90	4.72	5.48	4.74	5.77	4.75	3.97	4.09
Accuracy (%)^c^	98.11	96.87	99.42	99.99	102.55	108.38	103.72	100.58

^a^ n = 18; three days with six replicates per day.

^b^ Expressed as % RSD = (SD/mean) × 100.

^c^ Calculated as (mean determined concentration/nominal concentration) × 100.

### Stability

The predicted concentrations for LUME and 97–63 at 10 and 400 ng/mL samples deviated within the nominal concentrations in a battery of stability tests: AS (18 hrs), BT (6 hrs), repeated three FT cycles (FT-3) and at −70 ± 10°C for at least for 15 days (Table 3). The results were found to be within the assay variability limits during the entire process.

**Table 3:** **Stability of LUME and 97–63 in rat plasma**

	**LUME**				**97-63**			
	**Mean** ^**a**^	**SD**	**Precision** ^**b**^ **(%)**	**Accuracy** ^**c**^ **(%)**	**Mean** ^**a**^	**SD**	**Precision** ^**b**^ **(%)**	**Accuracy** ^**c**^ **(%)**
**10 (ng/mL)**								
0 hr (for all)	9.6	0.22	2.24	96.20	10.2	0.46	4.54	109.12
24 hrs (AS)	9.9	0.40	4.05	103.72	4.3	0.16	3.74	104.67
6 hrs (BT)	9.8	0.69	7.01	101.73	3.8	0.16	4.30	93.85
FT-3	9.6	0.74	7.74	99.48	3.9	0.13	3.34	98.16
15 days at −70°C	9.6	0.24	2.49	105.73	4.1	0.12	2.92	100.47
**400 (ng/mL)**								
0 hr (for all)	387.3	16.61	4.29	96.83	405.2	14.16	3.50	101.29
24 hrs (AS)	393.2	17.62	4.48	101.51	407.0	16.16	3.97	100.45
6 hrs (BT)	391.3	19.01	4.86	101.03	388.3	14.47	3.73	95.85
FT-3	413.5	12.34	2.98	106.78	404.3	6.15	1.52	99.79
15 days at −70°C	397.2	9.28	2.34	102.54	400.0	8.34	2.09	98.72

^a^ Back calculated plasma concentrations (n = 6).

^b^ Expressed as % RSD = (SD/mean) × 100.

^c^ Calculated as (mean determined concentration/nominal concentration) × 100.

### Dilution integrity

The percentage accuracy of diluted QCs was in the range of 94.56 to 107.68, while percentage precision values ranged from 0.89 to 7.52 for both the analytes. The results suggested that samples with concentrations greater than the upper limit of calibration curve could be re-analysed by appropriate dilution.

### Application to interaction study

**Figure 3:**
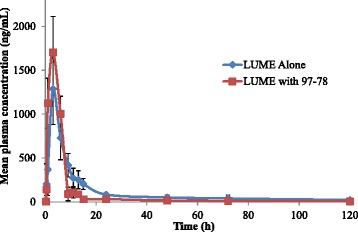
Linear plots of plasma concentration LUME after oral administration of LUME alone or in combination with 97–78 (means ± SD).

The rat plasma samples generated following administration of LUME and 97–78 were analysed by the newly developed and validated method along with QC samples. The mean plasma concentration-time profiles of LUME administered (10 mg/kg) alone or in combination with 97–78 (70 mg/kg) orally in rats, are shown in Figure 3. Table 4 summarizes the pharmacokinetic parameters of LUME and 97–78. The presence of 97–78 significantly (p <0.05) decreased the AUC_0-∞_ (63.26%) of orally administered LUME. Consequently, the RB% of LUME in the presence of 97–78 is remarkably decreased (64.41%) compared to the LUME alone. The T_max_ and C_max_ were not significantly altered by 97–78. LUME clearance (Cl/F) was increased by 64.84% and V_d_/F increased by 62.89% with 97–78 co-administration. As both apparent CL/F and apparent V_d_/F were increased almost proportionally, there was no significant effect on the t_1/2_ of LUME in presence of 97–78 (47.82 *vs* 46.94 hrs; p > 0.05).

**Table 4:** **The pharmacokinetic parameters of LUME and 97–78 in rats (n = 5 each)**

**Parameters**	**Control**		**LUME with 97-78**	
	**LUME (10 mg/kg)**	**97-78 (70 mg/kg)**	**LUME (10 mg/kg)**	**97-78 (70 mg/kg)**
**AUC**_**0-t**_ (h*μg/mL)	16.52 ± 2.96	9.10 ± 1.48	10.64 ± 0.71^*^	8.04 ± 1.26
**AUC**_**0-∞**_ (h*μg/mL)	17.42 ± 3.29	9.15 ± 1.46	11.02 ± 0.66^*^	8.15 ± 1.26
**C**_**max**_ **(μg/mL)**	1.81 ± 0.75	1.35 ± 0.17	1.92 ± 0.62	1.79 ± 0.69
**T**_**max**_ **(h)**	3.75 ± 1.50	2.2 ± 1.09	4 ± 2.45	0.5^#^
**V**_**d**_ **/F (L/kg)**	39.45 ± 6.26	106.05 ± 30.57	62.73 ± 8.40^*^	142.03 ± 28.13
**CL/F (L/h/kg)**	0.59 ± 0.09	7.81 ± 1.26	0.91 ± 0.06 ^*^	8.76 ± 1.38
**t**_**1/2**_ **(h)**	46.94 ± 6.52	9.27 ± 1.33	47.82 ± 6.48	11.25 ± 1.45
**RB%**	--	--	64.41^*^	88.35

All data are expressed as mean ± SD.

^*^P <0.05, *vs* LUME control; ^#^P <0.05, *vs* 97–78 control.

**Figure 4:**
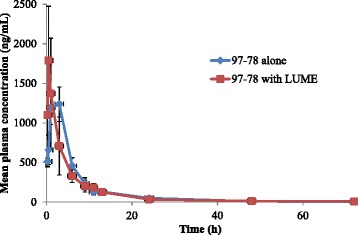
Linear plots of plasma concentration of CDRI 97–63 after oral administration of CDRI 97–78 alone or in combination with LUME (means ± SD).

Mean plasma concentration-time profiles of 97–78 are shown in Figure 4. The T_max_ of 97–78 was significantly decreased by LUME (2.2 *vs* 0.5 hrs; p < 0.05). LUME had no significant effect on other PK parameters of 97–78 (Table 4). Further studies are required to understand the mechanistic basis of alterations upon per-oral co-administration of anti-malarial as potential combinations. These results should be taken into consideration while designing clinical proof of concept pharmacodynamic studies and while designing dosage regimen.

## Conclusion

In this study, a highly sensitive, specific, reproducible, and high-throughput LC–ESI-MS/MS assay has been developed and validated to quantify LUME and CDRI 97–78 following protein precipitation extraction technique from rat plasma, for the first time using halofantrine as IS. Due to good sensitivity (LLOQ −3.9 ng/mL for both LUME and 97–63) of the assay and its short run time of 6 min, it offers a suitable platform for the determination of LUME and 97–63 in preclinical studies. The results of the interaction study show that there are potential chances of pharmacokinetic interactions between long-acting LUME and short-acting 97–78.

## Additional file

Additional file 1: Table S1. Recovery and matrix effect for LUME, 97–63 and IS in rat plasma.

## Abbreviations

LUME: Lumefantrine
ACT: Artemisinin combination therapy
IS: Internal standard
AS: Auto-sampler
BT: Bench top
FT: Freeze/thaw
LC-ESI-MS/MS: Liquid chromatography-electrospray ionization-tandem mass spectrometry
CMC: Carboxy methyl cellulose
MRM: Multiple reaction monitoring
LLOQ: Lowest limit of quantification
QC: Quality control
