# Supplementary material for: Simultaneous quantification of proposed anti-malarial combination comprising of lumefantrine and CDRI 97–78 in rat plasma using the HPLC–ESI-MS/MS method: application to drug interaction study
Source: Malar J. 2015 Apr 22;14:172. doi: 10.1186/s12936-015-0684-5 (PMC4429480; doi:10.1186/s12936-015-0684-5)
Supplement: Additional file 1: Table S1. — Recovery and matrix effect for LUME, 97–63 and IS in rat plasma. [file 12936_2015_684_MOESM1_ESM.docx]

**Additional file 1: Table S1:** Recovery and matrix effect for LUME, 97–63 and IS in rat plasma

| **Recovery** | | | | | | | |
| --- | --- | --- | --- | --- | --- | --- | --- |
|  | **LUME (ng/mL)** | | | **97-63 (ng/mL)** | | | **IS (ng/mL)** |
|  | 3.9 | 10 | 400 | 3.9 | 10 | 400 | 50 |
| Acetonitrile (%) | 98.38 ± 3.23 | 95.85 ± 0.94 | 96.06 ± 1.80 | 98.80 ± 2.54 | 102.34 ± 4.90 | 98.51 ± 3.54 | 96.32 ± 1.86 |
| Acidified acetonitrile (%) | 95.36 ± 2.47 | 96.37 ± 4.26 | 97.82 ± 1.96 | 89.47 ± 1.93 | 87.36 ± 1.07 | 81.82 ± 3.92 | 91.63 ± 4.41 |
| **Matrix effect** | | | | | | | |
| Acetonitrile (%) | -- | 8.70 ± 2.78 | 2.82 ± 1.39 | -- | 6.74 ± 3.27 | 4.39 ± 1.62 | 13.30 ± 4.32 |
